# Protocol for simultaneous profiling of transcription start sites and full-length transcripts from low-input samples using Smart-seq+5′

**DOI:** 10.1016/j.xpro.2026.104583

**Published:** 2026-05-26

**Authors:** Diego Rodriguez-Terrones, Marlies E. Oomen, Maria-Elena Torres-Padilla

**Affiliations:** 1 Institute of Epigenetics and Stem Cells, Helmholtz Munich, Munich, Germany; 2 Faculty of Biology, Ludwig-Maximilians Universität, Munich, Germany

**Keywords:** Genomics, RNAseq, Molecular Biology, Gene Expression

## Abstract

Transcriptomic approaches such as cap analysis of gene expression (CAGE) enable the identification of transcription start sites (TSS) and the quantification of promoter activity. However, these techniques require large RNA input amounts and cannot profile transcript bodies simultaneously. Here, we present a protocol for characterizing full-length transcripts while simultaneously identifying the precise location of TSSs using Smart-seq+5′, a low-input library preparation approach. We describe steps for RNA extraction with optional *in vitro* polyadenylation, reverse transcription and 5′ capture, Tn5 tagmentation for transcript coverage, and computational analysis. Based on the widely adopted Smart-seq2, Smart-seq+5′ offers improved sensitivity and can be employed to profile both polyadenylated and non-polyadenylated transcripts.

For complete details on the use and execution of this protocol, please refer to Oomen et al.^1^

## Highlights

• Instructions for transcription start site detection and full transcript coverage
• Steps for processing of low-input embryo samples and bulk sample types
• Guidance on experimental procedures and computational analyses
• Troubleshooting suggestions with concrete solutions during implementation

## Abstract (editor highlights)

Publisher’s note: Undertaking any experimental protocol requires adherence to local institutional guidelines for laboratory safety and ethics.

## Graphical abstract

**Figure:**
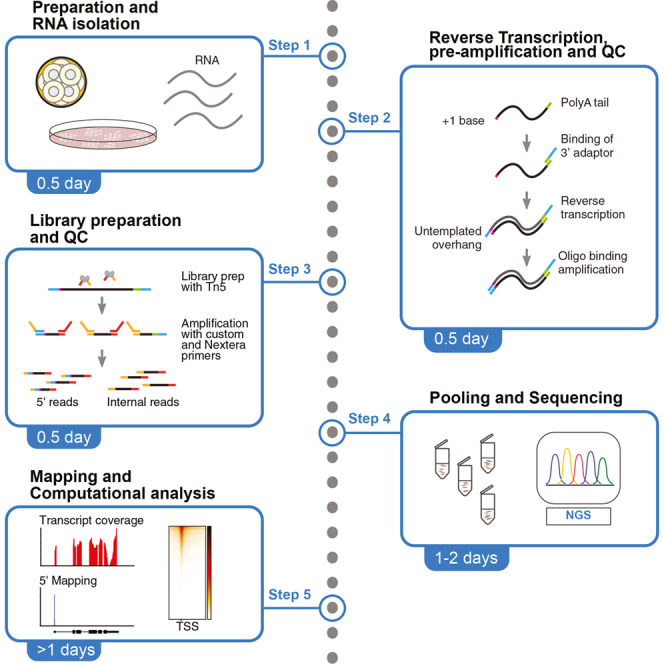

## Before you begin

Eukaryotic promoters are DNA sequences that facilitate the assembly of the transcriptional machinery and define sites of transcription initiation throughout the genome. These genomic regions can be predicted based on the presence of characteristic chromatin features such as H3K4me3 and accessible chromatin. Alternatively, they can be identified through transcriptomic approaches aimed at capturing the cap structure at the 5′ end of the transcripts driven by such promoters, thereby revealing the transcription start site (TSS) with base-pair resolution.

Compared to conventional transcriptomic methods, 5′ transcriptomic approaches offer additional layers of information by capturing the precise transcription start sites (TSSs) of RNA molecules. For instance, 5′ profiling provides insights into promoter architecture and facilitates the identification of alternative TSSs, enhancing our understanding of transcriptional regulation. This is particularly valuable when studying transposable elements (TEs) and other repetitive sequences since it enables the distinction between bona fide TE-driven transcription and genic read-through into TE-derived sequences.11^1^^,^^2^

Numerous approaches for profiling the 5′ ends of transcripts have been developed, employing a variety of molecular mechanisms for their capture. The pioneering Cap Analysis of Gene Expression (CAGE)^3^^,^^4^ and related approaches^5^ rely on the biotinylation of the cap structure and its subsequent purification to capture full-length capped transcripts. On the other hand, cap-dependent ligation approaches, such as Teloprime rely on the activity of a dsDNA-dependent ligase to preferentially ligate an adapter to molecules harboring a 5′ cap or terminal triphosphate. Given the complex workflows required by these techniques, more recent approaches have emerged that instead employ the template switching activity of the reverse transcriptase to incorporate an adapter at the cap site during reverse transcription, significantly streamlining the workflow. The use of template switching requires much smaller RNA inputs than previous approaches, thus enabling the generation of high-quality libraries from nanogram-scale RNA inputs^6^ and even single cells.^7^^,^^8^^,^^9^

Here, we describe Smart-seq+5′, an experimental and computational workflow to simultaneously achieve 5′ and full-length transcript coverage. Building upon Smart-seq2, Smart-seq+5′ implements molecular crowding during reverse transcription for greatly enhanced sensitivity and incorporates an optional *in vitro* polyadenylation step to enable profiling of non-polyadenylated transcripts such as histone genes. Furthermore, a modified tagmentation approach selectively captures transcript 5′ ends, in addition to the internal fragments derived from the transcript body. This allows for the quantification of full-length transcript bodies while in addition, determining transcription start sites with base-pair resolution. For an in-depth benchmarking of Smart-seq+5′ performance, we refer users to the relevant publications.^1^^,^^10^ A flow diagram of our method can be found in the graphical abstract as well as in our previous publication.^2^ Although we developed Smart-seq+5′ with a particular focus on the mammalian preimplantation embryo due to our scientific interests, Smart-seq+5′ is also applicable to other low input RNA samples, as we describe below.

### Innovation

Given that promoters are often cell-type specific, various efforts have focused on adapting template switching-based 5′ profiling approaches to single cells. In fact, one of the earliest single cell sequencing protocols – STRT-seq^7^^,^^8^^,^^11^ – was based on 5′ profiling. Perhaps because of the increased difficulty in optimizing reverse transcription conditions when incorporating barcodes in the 5′ adapter compared to the 3′ adapter, subsequent efforts such as SCRB-seq,^12^^,^^13^ Cel-seq^14^^,^^15^ and Drop-seq,^16^ all relied instead on 3′ barcoding, which would go on to become the standard approach. One exception was the widely adopted Smart-seq2, which offers full-length transcript coverage by dropping 5′ and 3′ barcoding altogether. Thanks to systematically optimized reverse transcription conditions, Smart-seq2 offers exceptional sensitivity and is perfectly tailored to mid-throughput experiments aimed at profiling tens to hundreds of samples. More recently, both Smart-seq3^17^^,^^18^ and FLASH-seq^19^ have incorporated 5′ barcoding with an emphasis on scalability to thousands of cells through the miniaturization of reaction conditions. Along similar lines, a commercial microfluidics-based 5′ profiling method is available through 10x Genomics, which is also aimed at capturing thousands of cells at low depth and therefore fails to efficiently capture low abundance transcripts, which is pertinent when investigating transposable element transcriptional activity.^2^^,^^20^ Thus, there is an open niche for a low-input 5′ profiling method that prioritizes sensitivity over throughput and that is amenable to rare or bulky samples – such as mammalian preimplantation embryos and biopsy material – that cannot be profiled using microfluidics approaches.

### Experimental design

**Timing: Variable**

Several experimental design decisions should be taken before starting this protocol.

1. Determine whether ERCC RNA spike-ins should be employed.

***Note:*** If downstream analyses require it, ERCC RNA spike-ins can be included in the lysis buffer. The normalisation against ERCC controls can be incorporated or not during the computational analyses and the use of ERCC does not interfere with the quality of the Smart-seq+5′ libraries. We recommend consulting a bioinformatician if it is unclear whether the downstream analyses require spike-in normalization. In the case of mammalian preimplantation embryos, in our experience relative normalization to the total counts is the most robust approach and is preferred over ERCC spike-in normalization. This is likely due to variations in absolute RNA content between embryos, which leads to noisier estimates in spike-in normalization compared to relative normalization. However, spike-in normalization is essential if absolute changes in RNA levels are of interest, and if that is the case, we encourage users to generate sufficient replicate samples to overcome the increased variance in ERCC-normalized expression level estimates. If this is the case, we strongly recommend preparing and sequencing a test sample to verify that ERCCs are incorporated at an adequate concentration, which typically amounts to ∼1–5% of the RNA input.

2. Determine whether *in vitro* polyadenylation should be employed.

***Note:*** The standard protocol only profiles polyadenylated transcripts because Smart-seq2 is based on the use of a poly(dT) oligo for initiating cDNA synthesis during reverse transcription. This precludes the detection of non-polyadenylated transcripts such as histone transcripts. An optional *in vitro* polyadenylation step thus allows capturing transcripts that are not polyadenylated endogenously. Incorporate the optional *in vitro* polyadenylation step if the research question requires profiling of such transcripts. Note that the addition of this step requires a ∼2x-fold increase in sequencing depth because of increased capture of rRNA transcripts.

3. Plan on collecting all samples in advance.

***Note:*** To minimize batch effects, it is strongly recommended to prepare all the libraries in a single batch, employing the same master mixes. When handling mammalian preimplantation embryo samples, we recommend collecting the single embryos in PCR strips. Samples can be collected in advance and stored at −80°C in lysis buffer until library preparation.

4. Include collection of spare samples for PCR cycle determination in experimental pan.

***Note:*** To determine the optimal PCR cycle number for preamplification, it is recommended to carry out the PCR cycle determination protocol (Preparation 5) on a spare set of representative samples spanning the RNA input range. Therefore, ensure that a few extra samples are collected in addition to the general sample collection described above. For example, if the dataset comprises a time course of preimplantation development between oocyte and 16-cell stage, PCR cycle determination could be carried out using 2x oocyte, 2 x 2-cell stage embryo and 2 x 16-cell stage embryo samples.

### Prepare lysis buffer aliquots

**Timing: 2 h**

Lysis buffer aliquots should be prepared in advance and stored at −80 °C until sample collection, to ensure that all collected samples undergo lysis under identical conditions. This is particularly critical if ERCC RNA spike-ins are used to prevent batch effects that can arise due to variability in spike-in.

5. Calculation of ERCC RNA spike-in dilution factor.
  a. Estimate the typical RNA content in the input samples.
    i. For mammalian preimplantation embryos, we have used the following estimates from the literature.^21^^,^^22^

**Table:** 

	Mouse	Cow	Pig
Oocyte Total RNA content	∼500 pg	980 pg	650 pg
    ii. For previously purified bulk total RNA, RNA content can be directly measured from the sample. See below for sample preparation of purified mRNA. Consider that the maximum and recommended input volume for such samples is 1 μL.
    iii. For other sample types, this should be estimated on a case-by-case basis.
  b. Estimate the typical mRNA content in the input samples.
    i. For mouse preimplantation embryos, the mRNA fraction of total RNA is estimated to amount to 5–8%.^23^
    ii. For most sample types, this is typically in the 1–5% range. We recommend the researcher to refer to literature resources for more accurate estimations of mRNA content in their sample type.
  c. Determine the target ERCC spike in concentration in the lysis buffer. For example, when aiming for a 1% ERCC spike-in concentration in mouse preimplantation embryos containing ∼500 pg total RNA, of which ∼6% is mRNA, the calculation is as follows:
    ERCCspike−in=inputtotalRNAxfractionmRNAxfractionERCC
    Example:
    ERCCspike−inRNAamount=500pgx0.06x0.01=0.3pg
    ***Note:*** The volume of lysis buffer employed in the Smart-seq+5′ protocol is 5.8 μL per sample for preimplantation embryo collection. Therefore, the concentration of ERCC RNA spike-ins in the lysis buffer can be calculated as follows:
    ERCCspike−inconcentration=ERCCspike−in/lysisvolume
    Example:
    ERCCspike−inRNAconcentrationinlysisbuffer=(0.3pg(fromstepc))/(5.8μL)=0.0517pg∕μL
  d. Calculate the dilution factor for the ERCC RNA spike-in stock. The stock solution (Invitrogen, 4456740) is provided at a concentration of 103 515.02 amol/μL or 29 819.53 pg/μL.
    DilutionFactor=ERCCstockconcentration/ERCCfinalconceration
    Example:
    DilutionFactor=(29.819.53pg/μL)/(0.0517pg/uL(fromstepd))=5.76511x

6. Before starting, ensure an RNase-free work environment. Prepare pipettes and surfaces by cleaning them with RNaseZap. Always employ filter tips.

7. Prepare lysis buffer aliquots for sample collection.
  a. If **not** using ERCC spike-ins:
    i. Mix the following reagents in a 1.5 mL or 5 mL Eppendorf tube. Scale up the volumes if necessary, depending on the number of samples:
    ii. **Note**: we recommend preparing the mastermix volumes with an excess of 20% to manage potential loss of volume when pipetting.

**Table:** 

	Per reaction
10x Clontech lysis buffer	0.58 μL
Nuclease free water	5.22 μL
Total	5.8 μL
    iii. Mix thoroughly by vortexing or using a P1000 set to at least 70% of the total volume.
    iv. Aliquot 5.8 μL of lysis buffer in PCR strip tubes. We recommend employing PCR strips harboring individual lids.
      ***Note:*** We recommend using PCR strips for sample collection, especially when handling embryo samples, as this allows for independent sample collection of different timepoints.
    v. Store the lysis buffer aliquots at −80 °C until sample collection.
  b. If using ERCC spike-ins:
    i. Prepare a 1:1000 dilution of ERCC RNA spike-ins in a 1 mL Eppendorf tube (1 μl of ERC RNA spike-in Mix into 999 μl of nuclease-free water).
    ii. Mix thoroughly using a P1000 set to at least 70% of the total volume.
    iii. Aliquot as 10 μL aliquots and store in −80 °C for later use.
    iv. To prepare ERCC-containing lysis buffer, mix the following reagents in a 1.5 mL or 5 mL Eppendorf tube. Scale up the volumes if necessary, depending on the number of samples:

**Table:** 

	Per reaction
10x Clontech lysis buffer	0.58 μL
Nuclease free water	variable
ERCC spike-in RNA 1:1000 dilution	variable
Total	5.8 μL
      The amounts above should be adjusted depending on the ERCC RNA spike-in dilution factor calculated above. For example, when employing the 576 511x dilution ratio for mouse preimplantation embryos, the volumes would be:
      **Table:** Example

Example for dilution factor 576 511x	Per reaction
10x Clontech lysis buffer	0.58 μL
Nuclease free water	5.21 μL
ERCC spike-in RNA 1:1000 dilution	0.01 μL
Total	5.8 μL
      ***Note:*** Make sure to prepare at least ∼20% more lysis buffer ERCC mastermix than the number of required reactions to manage any volume loss when pipetting.
    v. Mix thoroughly by vortexing or using a P1000 set to at least 70% of the total volume.
    vi. Using a P20, aliquot 5.8 μL of lysis buffer in PCR strip tubes. As above, we recommend employing PCR strips harboring individual lids.
    vii. Store the lysis buffer aliquots at −80 °C. Follow manufacturer’s recommendation for both lysis buffer and ERCC spike in regarding maximum storage time.

### Aliquot reagents

**Timing: 1 h**

We highly recommend aliquoted all reagents to ensure clean use and avoid unnecessary freeze-thaw cycles.

8. Prepare single-use 1 mL aliquots for the following reagents in RNAse-free 1.5 mL Eppendorf tubes:
  a. AMPure RNAClean XP magnetic beads
  b. AMPure DNA XP magnetic beads
  c. PEG-8000 40% solution
9. Store at 4 °C until use.

### Sample collection

**Timing: Variable**

When possible, all samples should be collected simultaneously to avoid unnecessary batch effects.

***Note:*** Before starting, ensure an RNAse-free work environment. Prepare pipettes and surfaces by cleaning them with RNaseZAP. Always employ filter tips.

10. If collecting mammalian preimplantation embryos:
  a. Isolate embryos as typically done for the species and stage required. In our experience it is not necessary to remove the zona pellucida.
  b. Wash embryos by transferring them to a drop of sterile, nuclease-free 1x PBS and wait for 2 min. Repeat twice more for a total of three washes.
  c. Using a fresh glass pasteur pipette, transfer a single embryo into the lysis buffer of a PCR strip tube (prepared as described in Preparation 2). Verify that the embryo was transferred by emptying the remaining pipette contents under the stereoscope to ensure that the embryo did not remain in the pipette. In addition to single embryos, this protocol can also be applied to pools of embryos. Reassess the optimal number of PCR cycles and if applicable ERCC spike-in ratios as described above when using a pool of embryos.
  d. Repeat until all embryos are collected. Ensure that a spare set of representative samples are collected for determining the number of PCR cycles needed for library preparation (as indicated in Preparation 5). Incubate the samples on ice while proceeding with the rest of sample collection.
  e. Store the lysed samples at −80 °C until library preparation.
11. If collecting *in vitro* cultured cells:
  a. Culture cells under appropriate conditions. Harvest cells and wash once in 1x PBS prior to RNA extraction.
  b. Extract RNA from cells using your preferred approach. It is critical that the extracted RNA exhibits good purity and integrity. Elute the extracted RNA in nuclease-free water. NOTE: We have successfully used the Reliaprep kit (Promega #Z6011) for this step, however any RNA extraction method that results in pure and high integrity RNA can be used.
  c. Verify RNA integrity by capillary electrophoresis. We recommend employing high quality RNA exhibiting a RIN value > 9. Lower RIN values can be employed at the risk of failing to capture longer transcripts. We do not recommend proceeding with samples with RIN values lower than 8.
  d. Measure RNA concentration by Nanodrop or Qubit and prepare a working dilution to 500 pg/μL in nuclease-free water. Diluted RNA samples can be stored at −80 °C until further processing for Smart-seq+5’. We recommend processing of samples within 6 months of collection.

### Estimate PCR cycle number

**Timing: 2–3 h**

To estimate the optimal number of PCR cycles for amplification, generate an amplification curve by performing a modified PCR reaction that includes a water-soluble fluorescent DNA intercalating dye, such as EvaGreen, as described below.

***Note:*** We strongly advise against employing DMSO-soluble dyes such as SYBR Green, as the DMSO will significantly affect the amplification curve.

***Note:*** We recommend this preparation step is performed for every new sample type.

12. Using a representative set of spare samples, proceed through the step-by-step protocol until Step 5c. Typically, 2–6 samples spanning the RNA input range are sufficient.
13. Thaw the necessary reagents on ice for 30 min
14. Prepare **MasterMix IV-B – Preamplification**:

**Table:** 

Reagent	Per reaction	Per reaction + 20% margin
KAPA HiFi 2x MasterMix	12.5 μL	15 μL
Nuclease-free water	1.25 μL	1.5 μL
ISPCR oligos (10 μM)	0.25 μL	0.3 μL
EvaGreen (20x in water)	1.00 μL	1.2 μL
**Total**	15 μL	18 μL

15. Retrieve reverse transcribed samples (Reverse transcription protocol, step 5) from thermocycler and place on ice.
16. Add 15 μL of **MasterMix IV-B** to each sample and mix thoroughly by pipetting.
17. Transfer the full reaction volume of each sample to individual wells of a qPCR plate and seal the plate.
18. Place the plate in a qPCR machine and launch the following program:

**Table:** 

Step	Temperature	Time	
Initial denaturation	98 °C	3 min	–
Denaturation	98 °C	20 sec	For 35 cycles
Annealing	67 °C	15 sec	
Extension	72 °C	6 min	
Final extension	72 °C	5 min	–
Hold	4 °C	Indefinite	–

19. Examine the amplification curve to select the optimal PCR cycle number for amplification. Identify the lowest PCR cycle number that is still within the exponential phase for all test samples. An example amplification curve for 2 replicate RNA samples derived from rabbit embryonic stem cells (rbESCs) is shown in Figure 1. Based on these curves we determined to use 15 PCR cycles for the preamplification PCR step for rbESCs samples.

**Figure 1:**
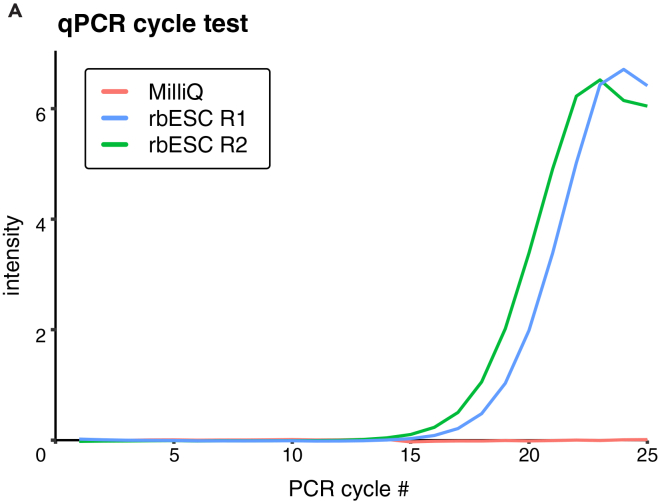
qPCR amplification curve of samples before tagmentation (A) Examples of two rabbit embryonic stem cell replicates and negative control (MilliQ).
20. Use the identified PCR cycle number for the preamplification PCR in step 6.

### Custom Nextera indices

**Timing: Variable**

***Note:*** To enable sequencing of full-length cDNAs using short-read sequencing platforms, it is necessary to fragment the cDNA into smaller segments. This is achieved using a Tn5 transposase-based method known as tagmentation, which simultaneously cleaves the cDNA and incorporates adapter sequences. These adapter sequences serve as primer binding sites for a subsequent PCR-based indexing step, allowing for sample multiplexing. Due to the random nature of Tn5 transposition, integration events can occur anywhere along a transcript. In the standard tagmentation approach, at least two transposition events within a given cDNA are necessary to produce the two primer binding sites required for successful amplification.

To specifically capture the 5′ ends of transcripts, Smart-seq+5′ employs a modified tagmentation strategy that incorporates a custom indexing primer designed to bind the template-switching oligonucleotide (TSO) sequence present at the 5′ end of the cDNA. This approach enables amplification between the TSO and an internal tagmentation site, requiring only a single transposition event for successful amplification. In contrast, within the body of the transcript, amplification occurs between pairs of internal tagmentation sites. Importantly, reads originating from the 5′ end can be identified based on the presence of the TSO sequence, allowing for the discrimination of 5′-derived fragments from internal ones. This enables the capture of transcription start site (TSS) information. Additionally, the reduced requirement of a single transposition event, rather than the typical two, enhances the sensitivity of the protocol for detecting short transcripts.^10^

Each sample requires three indexing primers: a standard P5 primer, a standard P7 primer, and a custom P7 primer. The standard P5 and P7 primers are used to amplify fragments that originate from within the transcript body, where tagmentation has occurred at two sites. The custom P7 primer, designed to bind the TSO sequence, is used in combination with the standard P5 primer to amplify fragments derived from the 5′ end of the transcript.

The standard Nextera indexing primers are commercially available from Illumina or IDT. On the other hand, the custom P7 primer targeting the TSO must be custom synthesized. To ensure correct demultiplexing of sequencing reads, the barcode sequence of the custom P7 adapter must match the barcode used in the standard P7 adapter for that sample. A set of 96 example standard and custom P5/P7 adapter sequences is provided in Table S1. Users have the option of either ordering the 3 full sets of adapter sequences according to this table, or to adjust the P7 index sequence of the custom P7 adapter to match the sequence of Nextera adapters that they already possess.

21. Pool the three indexing adapters should be at a final concentration of 2.5 μM each.

**CRITICAL:** Each sample should possess its own unique barcode set. We strongly recommend aliquoting this index pool into single use PCR plates to minimize the risk of cross-contamination. Each reaction requires 5 μL of this mix.

**Standard P5 adapter**

5’-

AATGATACGGCGACCACCGAGATCTACACNNNNNNNNTCGTCGGCAGCG∗T∗C-3′

**Standard P7 adapter:**

5’-

CAAGCAGAAGACGGCATACGAGATNNNNNNNNGTCTCGTGGGCTC∗G∗G-3′

**Custom P7 adapter:**

5′-CAAGCAGAAGACGGCATACGAGATNNNNNNNNGTCTCGTGGGCTCGG**AGATGTGTATAAGAGACAGAAGCAGTGGTATCAACGC∗A∗G**-3′

^1^The sample index is indicated as NNNNNNNN.

^2^The custom TSO binding sequence is indicated in **bold**.

^3^Asterisks indicate phosphorothioate bonds.

### Preparation of custom oligonucleotides

**Timing: Variable**

22. The following oligonucleotides must be procured, diluted to the appropriate concentration and aliquoted.
  a. Dilute Template switching oligo (TSO) to 100 μM in water.
      5′-AAGCAGTGGTATCAACGCAGAGTACATrGrG+G-3′
       ^1^ rG stands for RNA Guanine.
       ^2^ An LNA Guanine is indicated by +G.
  b. Dilute dT_30_V oligo to 10 μM in water.
      5′-AAGCAGTGGTATCAACGCAGAGTACT_30_V-3′
       ^1^ T_30_ stands for 30x thymine.
       ^2^ V stands for A/G/C.
  c. Dilute ISPCR oligo to 10 μM in water.
      5′-AAGCAGTGGTATCAACGCAGAGT-3′

### Prepare pipettes, surfaces, and reagents

**Timing: 1–2 h**

23. Before starting the protocol, ensure an RNAse-free work environment. Prepare pipettes and surfaces by cleaning them with RNaseZAP. Always employ filter tips.

***Note:*** This protocol uses magnetic beads for nucleic acid purification and therefore requires a magnetic rack compatible with PCR strip tubes. We use the DynaMag-96 (Thermo Fisher, Cat. No. 12331D); however, other models may also be suitable, provided they have side-facing magnets and sufficient magnetic strength to efficiently capture the beads.

24. To avoid batch effects, it is essential to prepare all samples using the same master mixes. We recommend preparing at least 20% excess volume to account for pipetting variability and ensure sufficient reagents for all samples.
25. For each protocol step, ensure that the right volumes of reagents are thawed for quick and efficient progression through the protocol.

## Key resources table

**Table:** 

REAGENT or RESOURCE	SOURCE	IDENTIFIER
**Chemicals, peptides, and recombinant proteins**		

10x Single-cell lysis buffer	Takara	635013
ERCC RNA Spike-In Mix	Invitrogen	4456740
AMPure RNAClean XP magnetic beads	Beckman Coulter	A63987
AMPure XP DNA magnetic beads	Beckman Coulter	A63881
E. coli Poly(A) Polymerase	NEB	M0276L
Superscript II RT	Invitrogen	18064014
DTT		
Betaine	Sigma	B0300-1VL
dNTP mix (10 mM)	Invitrogen	R0192
1 M MgCl_2_	Sigma	M1028
RNAse inhibitor	Takara	2313 A
PEG-8000 40% solution	Sigma	P1458-25ML
Kapa HiFi ReadyMix	Roche	KM2605
EvaGreen	Biotium	31000
Ethanol absolute	Generic	
Nuclease free water	Invitrogen	AM9937
RNAseZAP	Invitrogen	AM9780
PBS (nuclease-free)	Generic	

**Critical commercial assays**		

Kapa HiFi ReadyMix	Roche	KM2605
Nextera XT DNA Library preparation kit	Illumina	FC-131-1096
Qubit dsDNA HS Assay Kit	ThermoFisher Scientific	Q32854

**Oligonucleotides**		

dT30V Oligo	Preferred oligonucleotide supplier, HPLC purified	
TSO oligo	Preferred oligonucleotide supplier, HPLC purified	
ISPCR primers	Preferred oligonucleotide supplier, HPLC purified	
Illumina Nextera Unique Double Indexes (P5 + P7)	IDT/Illumina	
Smart-seq+5′ custom P7 Nextera index	Preferred oligonucleotide supplier, HPLC purified	

**Software and algorithms**		

FastQC	Babraham Institute	https://github.com/s-andrews/FastQC
Picard	Broad institute	https://github.com/broadinstitute/picard
Trimmomatic	Bolger et al.	https://github.com/usadellab/Trimmomatic
STAR	Dobin et al.	https://github.com/alexdobin/STAR
TEtranscripts	Jin et al.	https://github.com/mhammell-laboratory/TEtranscripts
Custom scripts	Torres-Padilla lab	https://github.com/meoomen/Smartseq5

**Other**		

Cooling racks		
Magnetic rack (DynaMag 96 side magnet)	ThermoFisher Scientific	12331D
PCR strips with individual lids	Sarstedt	72.991.002
Bioanalyzer/Fragment analyzer/TapeStation		
Qubit		
qPCR machine		
Nanodrop		
Thermocycler		

## Step-by-step method details

### RNA purification and optional polyadenylation

**Timing: 2 h**

The goal of this step is to extract and purify the RNA from cell or embryo lysates using AMpure RNA beads with the optional step of in vitro polyadenylation to capture all RNA.

1. Prepare reagents and mastermixes.
  a. Bring AMPure RNAClean XP magnetic bead aliquots to room temperature, i.e., 18–22°C (∼30 min). Account for at least 15 μL per sample if performing the standard protocol or 30 μL if performing *in vitro* polyadenylation.
  b. Prepare 40 mL of 80% ethanol in nuclease free water in a 50 mL falcon tube.
  c. Thaw the all reagents needed for this protocol step and optional in vitro polyadenylation on ice for 30 min. Once the reagents are thawed, vortex non-enzyme reagents and spin-down all reagents using a bench micro-centrifuge.
  d. Prepare **MasterMix I – Annealing.** Mix thoroughly and place on ice.
    **CRITICAL:** Volumes differ slightly depending on the starting material (samples stored in lysis buffer (e.g. embryo samples) vs. previously purified RNA). Ensure to follow the correct volumes accordingly.
    **MasterMix I-A – Annealing:** When starting from samples stored in lysis buffer or when starting from previously purified RNA samples and incorporating the optional in vitro polyadenylation step:

**Table:** 

Reagent	Per reaction	Per reaction + 20% margin
Nuclease-free water	0.95 μL	1.14 μL
RNAse inhibitor	0.05 μL	0.06 μL
dNTPs (10 mM)	1 μL	1.2 μL
dT_30_V oligo (10 μM)	1 μL	1.2 μL
**Total**	3 μL	3.6 μL
    **MasterMix I-B – Annealing:** When starting from previously purified RNA samples and not incorporating the optional *in vitro* polyadenylation step:

**Table:** 

Reagent	Per reaction	per reaction + 20% margin
RNAse inhibitor	0.05 μL	0.06 μL
dNTPs (10 mM)	1 μL	1.2 μL
dT30 oligo (10 μM)	1 μL	1.2 μL
**Total**	2.05 μL	2.46 μL
    e. if experimental design requires performing in vitro polyadenylation, prepare **MasterMix II – Polyadenylation.** Mix thoroughly and place on ice.
    **CRITICAL:** As above, volumes differ slightly depending on the starting material (samples stored in lysis buffer (e.g. embryo samples) vs. previously purified RNA). Ensure to follow the correct volumes accordingly.
    **MasterMix II-A – Polyadenylation**: When starting from samples stored in lysis buffer:

**Table:** 

Reagent	Per reaction	Per reaction + 20% margin
Nuclease-free water	3.5 μL	4.2 μL
E. coli Poly(A)merase 10x reaction buffer	0.5 μL	0.6 μL
RNAse inhibitor	0.25 μL	0.3 μL
ATP	0.5 μL	0.6 μL
E. coli Poly(A)merase	0.25 μL	0.3 μL
**Total**	5 μL	6 μL
    **MasterMix II-B – Polyadenylation**: When starting from previously purified RNA samples:

**Table:** 

Reagent	Per reaction	per reaction + 20% margin
Nuclease-free water	2.5 μL	3 μL
10x reaction buffer	0.5 μL	0.6 μL
RNAse inhibitor	0.25 μL	0.3 μL
ATP	0.5 μL	0.6 μL
E. coli Poly(A)merase	0.25 μL	0.3 μL
**Total**	4 μL	4.8 μL

2. RNA isolation from lysate.
  **CRITICAL:** If processing samples stored in lysis buffer, isolate RNA using AMPure RNAClean XP magnetic beads as indicated in this step. If starting from previously purified RNA and performing the optional *in vitro* polyadenylation step, skip step 2 and proceed directly to step 3. If starting from previously purified RNA but not performing the optional *in vitro* polyadenylation step, skip steps 2–3 and proceed directly to step 4.
  a. Using a cooling rack, collect samples from −80°C.
  b. Place samples at room temperature (∼18–22°C) for 1–2 min, then spin down briefly.
  c. Vortex AMPure RNAClean XP beads aliquots thoroughly.
  d. Add 15 μL of AMPure RNAClean XP beads to each sample and mix thoroughly by pipetting.
    ***Note:*** It is highly recommended to use a multichannel pipette to process an entire row of samples simultaneously.
  e. Incubate at room temperature for 5 min (∼18-22°C).
  f. Place tubes on magnetic rack and wait for 3–5 min.
  g. Set multichannel pipette to 20 μL and remove supernatant.
  h. Immediately add 100 μL of 80% ethanol per sample.
    i. Remove samples from the magnet and wash the beads gently by pipetting.
    ii. Place samples back on the magnet and wait at least 30 s.
      ***Note:*** Pipet onto the tube wall to minimize aerosols and bring down any beads that might have become stuck on the upper side of the tube.
  i. Thoroughly remove the ethanol without disrupting the beads.
    ***Note:*** Using a pipette set to 75 μL and removing the supernatant twice will result in the full removal of all ethanol from the beads.
  j. If the optional *in vitro* polyadenylation step is to be skipped, proceed directly to step 4a.

3. If experimental design requires performing *in vitro* polyadenylation:
  a. If proceeding from step 2: without letting the pellets dry (no cracking!), add 5 μL of **MasterMix II-A – Polyadenylation** to each tube and re-suspend beads by pipetting.
    If starting from previously purified RNA: add 4 μL of **MasterMix II-B – Polyadenylation** to 1 μL of RNA sample.
    ***Note:*** Given the small volumes to pipette, it might be more convenient to use a single channel pipette than a multichannel pipette for this step, especially given the use of the magnetic beads.
  b. Incubate samples in a thermocycler as follows:

**Table:** 

Step	Temperature	Time
*In vitro* polyadenylation	37 °C	5 min
  c. Purify RNA again as in steps 2c-i, then proceed to step 4.

4. Annealing.
  a. If proceeding from steps 2 or 3: without letting the pellets dry (no cracking!), add 3 μL of **MasterMix I-A – Annealing** to each tube and re-suspend beads.
    If starting from previously purified RNA and omitting the optional *in vitro* polyadenylation step: add 2 μL of **MasterMix I-B – Annealing** to 1 μL of RNA sample.
    ***Note:*** Given the small volumes to pipette, it might be more convenient to use a single channel pipette than a multichannel pipette for this step.
  b. Incubate in a thermocycler at:

**Table:** 

Step	Temperature	Time
Annealing	72 °C	3 min
Hold	4 °C	Indefinite
  c. Meanwhile, prepare **MasterMix III – Reverse transcription**:

**Table:** 

Reagent	Per reaction	per reaction + 20% margin
Superscript II 5x RT buffer	2 μL	2.40 μL
Betaine (5 M)	2 μL	2.40 μL
PEG-8000 solution (40%)	1.6 μL	1.92 μL
DTT	0.5 μL	0.6 μL
RNAse inhibitor	0.25 μL	0.3 μL
TSO	0.1 μL	0.12 μL
MgCl_2_ (1 M)	0.06 μL	0.072 μL
Superscript II RT	0.5 μL	0.06 μL
**Total**	7 μL	8.4 μL
    ***Note:*** Add reagents in the listed order. Mix thoroughly by pipetting before and after addition of RT. Note that the 40% PEG solution is highly viscous and should be pipetted carefully and slowly.

### Reverse transcription and pre-amplification

**Timing: 4 h**

In this step the RNA is reverse transcribed to acquire cDNA molecules, followed by amplification of the cDNA library.

5. Reverse transcription.
  a. Retrieve samples from thermocycler and place on ice.
  b. Add 7 μL of **MasterMix III – Reverse transcription** to each sample and pipet up and down until thoroughly mixed.
    ***Note:*** This mix is viscous and should be mixed carefully.
  c. Incubate in a thermocycler using the following program:

**Table:** 

Step	Temperature	Time
Reverse transcription	42 °C	90 min
Inactivation	70 °C	15 min
Hold	4 °C	Indefinite

6. Pre-amplifcation.
  a. Thaw the reagents necessary for the pre-amplifcation step on ice 30 min before the end of the RT program:
  b. Equilibrate the AMPure XP DNA beads to room temperature (∼18–22°C), ∼30 min). In addition, prepare 40 mL of 80% ethanol in nuclease free water in a 50 mL falcon.
  c. Prepare **MasterMix IV – Preamplification**:

**Table:** 

Reagent	Per reaction	per reaction + 20% margin
KAPA HiFi 2x ReadyMix	12.5 μL	15 μL
Nuclease-free water	2.25 μL	2.7 μL
ISPCR oligos	0.25 μL	0.3 μL
**Total**	15 μL	18 μL
  d. Retrieve samples from thermocycler and place on ice.
  e. Add 15 μL of **MasterMix IV – Preamplification** to each sample
    i. Mix thoroughly by pipetting.
  f. Incubate in a thermocycler using the following program:

**Table:** 

Step	Temperature	Time	
Initial denaturation	98 C	3 min	–
Denaturation	98 C	20 sec	For X cycles (see Preparation 5)
Annealing	67 C	15 sec	
Extension	72 C	6 min	
Final extension	72 C	5 min	–
Hold	4 C	Indefinite	–

7. cDNA purification and clean up.
  a. Retrieve samples from thermocycler and place on ice.
  b. Vortex AMPure XP DNA beads thoroughly.
  c. Add 12.5 μL of AMPure XP DNA beads per sample.
    i. Mix thoroughly by pipetting.
  d. Incubate at room temperature for 5 min (∼18–22°C).
  e. Meanwhile, prepare new PCR strips to store final eluate.
  f. Place tubes on the magnetic rack and wait for 1–2 min.
  g. Set multipipette to 35 μL and remove supernatant.
  h. Immediately add 200 μL of 80% ethanol per sample.
  i. Resuspend the beads gently by pipetting and wait 30 s.
    ***Note:*** If you pipet onto the tube wall, you can minimize aerosols and bring down any beads that might have become stuck on the upper side of the tube.
  j. Aspirate residual supernatant with a pipette
    ***Note:*** Make sure to fully remove all residual ethanol. It can help to set to 150 μL and pipette to remove supernatant twice.
  k. Without letting the beads dry (no cracking!), add 17.5 μL of elution buffer.
    i. Remove the samples from the magnetic rack and re-suspend beads.
  l. Incubate for 5 min at room temperature (∼18–22°C).
  m. Place tubes on the magnet for 2 min.
  n. Transfer 15 μL of supernatant to a fresh PCR strip.
    **Pause point:** Samples can be stored at −20 °C until further processing.

### Quality control

**Timing: 2–4 h**

This step assesses quality, concentrationand integrity of the cDNA by Qubit and capillary electrophoresis prior to further processing.

**Figure 2:**
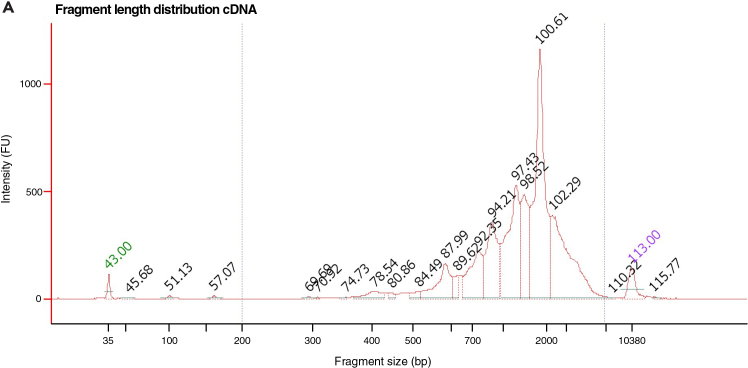
Fragment length distribution of an amplified Smart-seq+5′ sample prior tagmentation (A) Example shown originates from a single rabbit embryo.

8. Quantify concentration and assess cDNA integrity.
  a. Quantify the concentration of each sample using the Qubit High Sensitivity dsDNA assay kit.
    ***Note:*** For single mammalian preimplantation embryos, we typically observe cDNA concentrations between 500 pg / μL and 10000 pg / μL.
  b. Evaluate the integrity and size distribution of the amplified cDNA using capillary electrophoresis.
    ***Note:*** Suitable instruments include the Agilent Bioanalyzer, Fragment Analyzer, or TapeStation. Ensure that the assay used is compatible with the concentration range of your samples. During analysis, adjust the integration window to accurately capture the cDNA fragment distribution, and record both the median fragment size and the overall concentration. In our experience, median fragment lengths typically fall between 1,400 and 2,000 bp (see Figure 2).
    **CRITICAL:** Primer dimers may manifest as a sharp peak at 50–100 bp and must be eliminated by size selection before proceeding to Step 6 if they occur in significant amounts. If samples do not pass QC, additional clean up steps can be done. More information can be found in the troubleshooting section.

### Library preparation

**Timing: 4 h**

In this step the cDNA library will be tagmented using transposase. Following a clean up, the final Smart-seq+5′ library will be amplified to generate the library ready for sequencing.

9. Tagmentation reaction.
  a. Transfer 2 μL of each sample to a fresh 96-well plate or PCR strips.
  b. Dilute each sample to 120 pg/μL by adding nuclease-free water.
    ***Note:*** Dilution ratios should be estimated for each sample individually, and volumes will differ per sample. Pipet 2.5 μL of each sample (120pg/μL) per sample into PCR strips and place on ice or in a cooling rack.
    **CRITICAL:** Ensure accurate dilution and pipetting as the tagmentation reaction is extremely sensitive to the DNA input amount.
  c. Equilibrate the AMPure XP DNA beads to room temperature (∼18–22°C), ∼30 min.
    **CRITICAL:** At least 30.5 μL is required per sample. In addition, at least 200 μL of 80% ethanol is required per sample.
  d. Thaw the tagmentation reagents on ice and preheat the thermocycler to 55C.
  e. Once the reagents are thawed, vortex non-enzyme reagents and spin down all reagents.
  f. Prepare **MasterMix V – Tagmentation**:

**Table:** 

Reagent	Per reaction	Per reaction + 20% margin
Tagment DNA buffer	5 μL	6 μL
Amplicon Tagment Mix	2.5 μL	3 μL
**Total**	7.5 μL	9 μL
  g. Using a multichannel pipette, add 7.5 μl of **MasterMix V – Tagmentation** to each sample and pipet until homogeneous.
    ***Note:*** Keep PCR strip on ice or in a cooling rack.
  h. Transfer samples to the thermocycler and incubate for 5 min.

**Table:** 

Step	Temperature	Time
Tagmentation	55 °C	5 min
Hold	4 °C	Indefinite

10. Amplification of tagmented library
  a. Retrieve samples from thermocycler and place on ice
  b. Using a multichannel pipette, add 2.5 μL of NT buffer to each sample and mix thoroughly by pipetting.
  c. Incubate for 5 min at room temperature (∼18–22°C).
  d. Using a multichannel pipette, add 5 μL of Nextera index mix to each sample.
    **CRITICAL:** Ensure this index mix contains all 3 indexes described in Preparation 6 at a final concentration of 2.5 μM. It is not necessary to mix at this point.
  e. Add 7.5 μl of Nextera PCR mix (NPM) to each sample and mix thoroughly by pipetting.
    i. Place tubes on ice or in cooling rack after addition of NPM mix.
  f. Incubate in a thermocycler using the following program:

**Table:** 

Step	Temperature	Time	
Gap filling	72 °C	3 min	–
Initial denaturation	95 °C	30 sec	–
Denaturation	95 °C	10 sec	For 12 cycles
Annealing	55 °C	30 sec	
Extension	72 °C	30 sec	
Final extension	72 °C	5 min	–
Hold	4 °C	Indefinite	–

11. Clean up of amplified library.
  a. Retrieve samples from thermocycler and place on ice.
  b. Vortex AMPure XP DNA beads thoroughly.
  c. Add 15 μL of AMPure XP DNA beads per sample and mix thoroughly by pipetting.
  d. Incubate at room temperature for 5 min (∼18–22°C).
  e. Meanwhile, prepare and label 3x sets of PCR strips.
    ***Note:*** Two will be used in the following cleanup steps and one additional one will be used for final collection.
  f. Place tubes on magnetic rack and wait for 5 min.
  g. One PCR strip at a time:
    i. Set multichannel pipette to 100 μL and remove supernatant.
    ii. Immediately add 100 μL of 80% ethanol per sample. Resuspend the beads gently by pipetting and wait at least 30 s.
    iii. Aspirate residual supernatant with a pipette
      ***Note:*** As above, it can be easier to aspirate 75 μL twice to ensure full removal of the ethanol
    iv. Without letting the beads dry (no cracking!), add 12.5 μL of elution buffer and re-suspend beads.
    v. Place at room temperature (∼18-22°C) away from the magnet and proceed to the next PCR strip.
  h. Incubate each PCR strip away from the magnet for at least 5 min.
    i. Place tubes on the magnet for 2 min.
    i. Transfer 10 μL of supernatant to a fresh PCR strip.
    ii. Discard PCR strip with leftover beads.
  i. Add 5.5 μL of AMPure DNA beads to each sample and mix thoroughly by pipetting.
  j. Incubate at room temperature for 5 min (∼18–22°C).
  k. Place tubes on magnetic rack and wait for 5 min.
  l. Transfer 13 μL of supernatant to a fresh PCR strip.
  m. Add 10 μL of AMPure DNA beads to each sample and mix thoroughly by pipetting.
  n. Incubate at room temperature for 5 min (∼18–22°C).
  o. Place tubes on magnetic rack and wait for 5 min.
  p. One PCR strip at a time:
    i. Set multichannel pipette to 20 μL and remove supernatant.
    ii. Immediately add 100 μL of 80% ethanol per sample. Resuspend the beads gently by pipetting and wait at least 30 s.
    iii. Aspirate residual supernatant with a pipette.
    iv. Without letting the beads dry (no cracking!), add 12.5 μL of elution buffer and re-suspend beads.
    v. Place at room temperature (∼18–22°C) away from the magnet and proceed to the next PCR strip.
  q. Incubate each PCR strip away from the magnet for at least 5 min.
  r. Transfer 10 μL of final eluate to a fresh PCR strip.
    **Pause point:** Samples can be stored at −20 °C until quality control.

### Quality control of amplified libraries

**Timing: 2**–**4 h**

Prior to sequencing, this step aims to assess the quality, integrity and concentration of the final Smart-seq+5′ library prior to sequencing.

**Figure 3:**
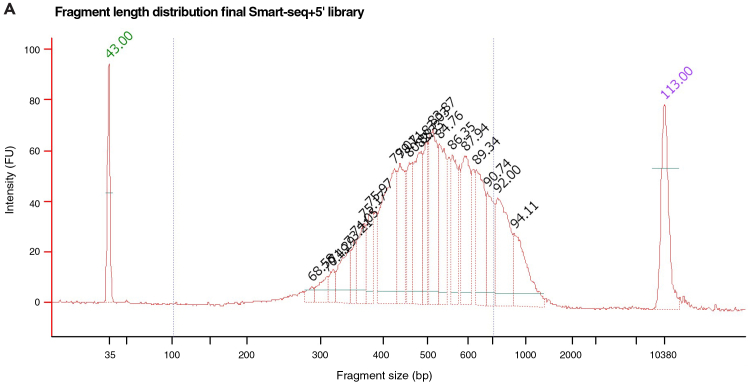
Fragment length distribution after tagmentation and final amplifciation (A) The sample shows the final Smart-Seq+5′ library from a single rabbit embryo.

12. Quantify concentration and assess DNA integrity.
  a. Quantify the concentration of each sample using the Qubit High Sensitivity dsDNA assay kit.
    ***Note:*** For single mammalian preimplantation embryos, we typically observe DNA concentrations between 500 pg / μL and 3000 pg / μL.
  b. Evaluate fragment size distribution of the library using capillary electrophoresis.
    ***Note:*** Suitable instruments include the Agilent Bioanalyzer, Fragment Analyzer, or TapeStation. Ensure that the assay used is compatible with the concentration range of your samples. During analysis, adjust the integration window to accurately capture the entirety of the fragment distribution, and record both the median fragment size and molarity. In our experience, median fragment lengths typically fall between 300 and 500 bp, while molarity ranges from 1000 to 10000 pM. (see Figure 3)
    **CRITICAL:** If considerable primer dimers or a significant fraction of fragments above 800 bp remain, additional size selection might have to be performed using magnetic beads. See troubleshooting section for additional information.

### Pooling of sequencing libraries

**Timing: 2 h**

Using the concentration as measured above, multiple libraries will be pooled in preparation for sequencing.

13. Prepare sequencing pools.
  a. Consult your NGS provider for sample submission guidelines.
    ***Note:*** A typical Illumina library should be submitted at a minimum concentration of 2.5 nM in a minimum volume of 30 μL.
  b. If equal sequencing depth is desired for each sample, pool equimolar amounts of each sample.
    **CRITICAL:** Ensure the final pool adheres to the volume and concentration guidelines of the NGS provider.

### Sequencing

**Timing: Variable**

Pooled Smart-seq+5′ libraries are sequenced on a next generation sequencing (NGS) platform.

14. Sequence libraries using NGS platform.

**CRITICAL:** Libraries should be sequenced using paired-end Illumina sequencing. We strongly recommend a 2 × 150 bp read configuration, as longer reads improve the fraction of uniquely mappable sequences.

***Note:*** For single mammalian preimplantation embryos, we recommend a minimum sequencing depth of 5 million read pairs per sample to ensure adequate transcriptome coverage.^2^ If performing polyadenylation, we recommend at least 10 million read pairs per sample given the higher rRNA capture.

### Computational processing of Smart-seq+5′ data

**Timing: Variable**

Smart-seq+5′ data is processed and analyzed

***Note:*** All software needed for analysis of Smart-seq+5′ data is listed in the key resources table. We recommend using conda to install and manage software. You can find instructions to download all needed software as conda environments on our github page (https://github.com/meoomen/Smartseq5/tree/main/conda_envs).

***Note:*** Detailed instructions on the commands used for processing of Smart-seq+5′ data can also be found on github (https://github.com/meoomen/Smartseq5/). A complete example of our recommended workflow can be found in the script “runRNASeqProcessingPE.sbatch”.

15. Classify sequencing data into 5′ and internal fragments.
  a. Run fastQC of all sequenced libraries to assess library quality prior to starting the analysis.
  b. Trim Illumina Nextera adaptors from sequence reads using trimmomatic.
    >trimmomatic PE Read1.fq.gz Read2.fq.gz -baseout ${id}.clean.fastq.gz -threads ${cores} ILLUMINACLIP:NexteraPE-PE.fa:2:30:10:1:true MINLEN:30
    **Critical:** Only keep paired reads that are a minimum length of 30bp each.
    ***Note:*** You can find a fasta file containing the sequences of Illumina Nextera adaptors on our github page (https://github.com/meoomen/Smartseq5/tree/main/adaptors). Do not trim Smart-seq adapters at this point since they will be used to classify reads as 5′ or internal.
  c. Classify reads into 5′, 3′ or internal reads.
    i. Align the 5′ and 3′ adapter sequences to the P7-derived read (Read 2 in data generated in Illumina NovaSeq sequencers) and select the one with the best score.
    ii. Classify reads not containing an adapter sequence as internal reads.
      ***Note:*** We provide a multithreaded implementation of this workflow on our github in the following scripts: "launchTailedRNAseqProcessing.sh" and "processTailedRNAseq.py".
      ***Note:*** 5′ fragments will be identified based on the adapter sequence “AAGCAGTGGTATCAACGCAGAGTAC**ATGGG**” while 3′ fragments will be identified based on the adapter sequence “AAGCAGTGGTATCAACGCAGAGTAC**TTTTT**”. Note that 3′ reads occur only at a very low frequency and are not efficiently captured.
      **CRITICAL:** These scripts will produce three fastq files: one containing 5′ fragments, another one containing internal fragments and a third one containing 3′ fragments. These read sets should be processed separately in steps 15 and 16.

16. Align internal fragments
  ***Note:*** Internal fragments can be processed as standard non-stranded paired-end RNA-seq reads. We recommend filtering out multimapping reads and if interested, carrying out TE-centric analyses with the 5′ fragments instead.
  a. Align **internal reads** using STAR to the genome of interest. If ERCC spike-in molecules were used, these sequences should be appended to the genome fasta file in order to be mapped.
  b. Remove all secondary alignments, supplementary alignments and unmapped reads using samtools.
    ***Note:*** Multimapping reads can be omitted using the -q 255 flag if desired.
    > samtools view -q 255 -F 2308 -o Output.bam Input.bam
    ***Note:*** We strongly recommend removing reads mapping to rRNA loci. This is particularly important for libraries employing *in vitro* polyadenylation. We provide an example workflow on our github page in the file “run_rRNA_removal.sbatch”. In brief, bedtools intersect is used to identify reads aligning to rRNA loci, samtools view is used to generate a read name list to exclude, and picard FilterSamReads is used to exclude reads from the input bam file. A bed file containing rRNA loci can be generated from a repeatmasker track.
    >bedtools intersect -a Input.bam -b rRNA_coords.bed -sorted -g chrom_sizes.txt > rRNAreads.bam
    >samtools view rRNAreads.bam | awk '{print $1}' | sort | uniq > filter.txt
    >picard FilterSamReads --QUIET true -I Input.bam -O Output.norRNA.bam --READ_LIST_FILE filter.txt --FILTER excludeReadList
  c. Sort and index bam files using samtools.
    >samtools sort -o Output.bam Input.bam
    >samtools index Input.bam
    ***Note:*** The resulting bam file can be used for standard gene centric analyses such as gene expression quantification using featurecounts.^24^
    ***Note:*** Bigwig files can be computed using a combination of bedtools genomecov and bedGraphToBigWig. These can be used to investigate transcript body coverage and for visualization.

17. Align 5′ fragments.
  ***Note:*** Reads containing the TSO sequence have been classified as 5′ fragments in step 14. These reads should be processed separately from the internal reads since they require special considerations. For instance, in contrast to the internal reads, the 5′ reads are stranded since the presence of the adapter sequence allows us to disambiguate the strand from which they derive. In addition, the transcription start site corresponds to the first aligned base of read 2. While these reads should be aligned as paired-end reads for optimal accuracy, read 1 can be discarded afterwards since it is non-informative for TSS analyses.
  a. Align reads using STAR to the genome of interest.
    **CRITICAL:** If ERCC spike-in molecules were used, these sequences should be appended to the genome fasta file in order to be mapped.
    ***Note:***--outSAMprimaryFlag AllBestScore can be used if downstream TE analyses required it. This will ensure all multimapping reads with the same best score to be considered primary alignments, meaning that true multimappers will be maintained in the output bam file.
  b. Remove all secondary alignments, supplementary alignments and unmapped reads using samtools. This produces a bam file containing uniquely mapping and multimapping alignments.
    >samtools view -F 2308 -o Output.bam Input.bam
    ***Note:*** We typically remove reads mapping to rRNA loci. This is particularly important for libraries employing *in vitro* polyadenylation. We provide an example workflow on our github page in the file "run_rRNA_removal.sbatch".
  c. In brief:
    i. use bedtools intersect to identify reads aligning to rRNA loci.
    ii. samtools view to generate a read name list to exclude.
    iii. picard FilterSamReads to exclude reads from the input bam file.
      ***Optional:*** A bed file containing rRNA loci can be generated from a repeatmasker track.
      >bedtools intersect -a Input.bam -b rRNA_coords.bed -sorted -g chrom_sizes.txt > rRNAreads.bam
      >samtools view rRNAreads.bam | awk '{print $1}' | sort | uniq > filter.txt
      >picard FilterSamReads --QUIET true -I Input.bam -O Output.norRNA.bam --READ_LIST_FILE filter.txt --FILTER excludeReadList
  d. For processing of 5′ fragments, keep only Read2 using samtools.
    >samtools view –f 128 -o FivePrimeFragments.read2.bam FivePrimeFragments.bam
    ***Note:*** 5′ fragments are captured in a stranded fashion, so if interested in strand directionality, bigwig files can be generated from bam files as plus and minus strand separately using samtools as follows:
    >samtools view -f 16 -o MinusStrand.bam Input.bam
    >samtools view -F 16 -o PlusStrand.bam Input.bam
  e. Split bamfiles for uniquely mapping and unique+multimapping reads using samtools.
    >samtools view –q 255 -o Output.unique.bam Input.bam
    ***Note:*** The bam file containing exclusively uniquely mapping reads can be used for non-multimapping aware analyses. The original bam file containing both uniquely mapping and multimapping reads can be used for TE-centric analyses if supported by the tool.
    ***Note:*** Only use multimapping reads when the downstream tool explicitly supports them. For example, use the unique + multimapping reads for TE quantification with TEcount, but only use unique mapping reads when generating bigWig files with bedtools genomecov.
  f. Compute bigWig genome tracks using a combination of bedtools genomecov and bedGraphToBigWig with the bam file containing uniquely mapping reads as input
    ***Note:*** These can be used to investigate transcript coverage or to visualize TSS architecture using tools such as deeptools.^25^ For most visualizations of 5′ reads, we recommend considering only the 5′ coordinate of read 2, as this constitutes the site of transcription initiation with base-pair resolution.
  g. Quantify TE counts using TEcount^26^ using unique and multimapping reads. **Note:** For accurate TE quantification, we recommend using only 5′ transcripts, read 2 as input for TEcount.

## Expected outcomes

### Endpoint PCR cycle determination

In this preparation, the optimal number of PCR cycles for preamplification of full length cDNAs is determined. In our experience, libraries typically require 10-18 PCR cycles of preamplification. In the case of mammalian preimplantation embryos, we have successfully produced libraries using 14 preamplification cycles.

### Quality control after full-length cDNA preparation (Step 8)

The expected fragment size distribution after preamplification can be seen in Figure 2. The average fragment size should be 1.5–2 kbp and there should be few fragments smaller than 500bp.

### Quality control after tagmentation (Step 12)

After tagmentation and final amplification, the fragment size distribution should span between 300 and 800 bp (as seen in Figure 3).

### Bioinformatic analyses (step 15–17)

Following mapping and processing Smart-seq+5′ libraries, we recommend computing the following quality control statistics: total number of reads sequenced per sample; total number of reads after adapter trimming, quality filtering and minimum read length filtering; proportion of reads mapping to rRNA loci; proportion of uniquely mapping and multimapping reads; number of genes detected; if employing spike-in RNAs, proportion of reads mapping to ERCC spike ins; if employing *in vitro* polyadenylation, number of reads mapping to non-polyA transcripts such as canonical histone genes. Quality of libraries can be assessed by the following library characteristics; total number of sequenced reads (>1,000,000 reads), total number of valid reads after adapter trimming, mapping and removal of rRNAs (>0.5 million valid reads), number of genes detected (>10000 genes detected in a single embryo or replicate).

### Quality control of 5′ capture

**Figure 4:**
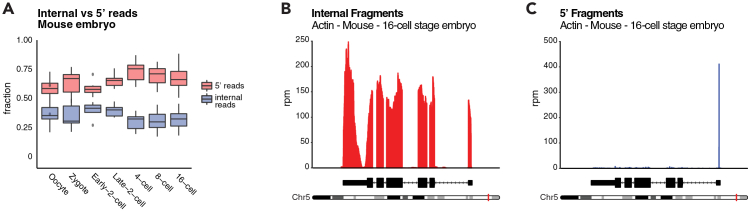
Example of Smart-Seq+5′ analysis results (A) Ratio of 5′ over internal fragments in Smart-seq+5′ libraries of mouse preimplantation embryos. The boxplots indicate the mean with limits at upper and lower quartile analyzed per stage. (B and C) Smart-seq+5′ internal fragments (B) and 5′ fragments (C) mapped to the mouse actin gene. Data shown originate from the mouse 16-cell stage embryo.

If ERCC RNA spike-ins are employed, they can be used to assess the efficiency of 5′ capture (Figure 4A). Note that while they serve as a reference ground truth given their synthetic nature, their 5′ end is not capped but rather phosphorylated and therefore they are an imperfect proxy for cellular transcripts. Furthermore, note that the reference sequences for ERCC spike-ins available from ThermoFisher are missing a short region at the 5′ end that must be manually corrected before alignment. As an alternative, known housekeeping genes such as β-actin (*Actb*) can be used to check for proper TSS capture and full-length transcript coverage (Figures 4B and 4C). Examples of genes and transposable element sequences with low abundance transcripts can be found in our previous publications.^2^^,^^20^ We recommend reviewing: proportion of 5′ and internal reads (Figure 4A); proportion of 5′ reads at annotated promoters (as shown in Figure S1 in Oomen, Rodriguez-Terrones et al;22^2^; and proportion of 5′ reads at 5′ end of ERCC spike-ins (as shown in Figure S1 in Oomen, Rodriguez-Terrones et al;22^2^).

## Limitations

In contrast to alternative 5′ capture approaches such as Smart-seq3 or FLASH-seq, Smart-seq+5′ requires custom Nextera indices in order to capture the 5′ end.

The template switching oligo (TSO) is known to misprime at rRNA and produce a significant fraction of rRNA-derived reads in mouse. Other mispriming events can occur depending on the species. Depending on the severity, alternative adapter sequences might need to be employed.

## Troubleshooting

### Problem 1

Short fragments are present after cDNA preamplification (Step 8). This can be the result of either degraded sample RNA or when reverse transcription reagents are contaminated with RNAses. Alternatively, a distinct peak of short fragments around 50-100bp can be caused by primer dimers.

### Potential solution

Repeat the protocol with new reagents or a new batch of samples to identify the source of the degradation. If the sample is the culprit, carefully collect samples again avoiding sources of RNAse contamination. It is recommended to flash freeze immediately after collection. If the short fragments are caused by primer dimers, this must be completely eliminated by size selection before proceeding to tagmentation (step 7).

### Problem 2

Fragment size distribution is too high after tagmentation (Step 12). Majority of the library is not between 300 and 800 bp, potentially caused by using too much DNA in the tagmentation reaction.

### Potential solution

Minor deviations from this distribution can be adjusted by an additional round of size selection. In severe cases, it might be necessary to modify the DNA input amount used in the tagmentation reaction (Step 9). Lowering the DNA input amount will result in a decrease in average fragment size, while increasing the input amount will result in an increase in average fragment size. It is critical to remove by size selection all traces of primer dimers, if present.

### Problem 3

Fragment size distribution is too small after tagmentation (Step 12). Majority of the library is not between 300 and 800 bp.This can be the result of using too little DNA in the tagmentation reaction.

### Potential solution

Increase DNA input in tagmentation (Step 9).

### Problem 4

Large amount of primer dimers are present after tagmentation (Step 12) due to inadequate size selection.

### Potential solution

perform an additional size selection step to remove primer dimers (Step 11).

### Problem 5

Excessive amount of ERCC spike-in molecules are present in library after sequencing (>10–20%) (Step 15–16). This can be the result of using a ERCC spike-in concentrations that was too high.

### Potential solution

Estimate a refined ERCC spike-in concentration based on sequencing results and repeat protocol. Carry out a trial sample again to confirm suitability of new concentration (Preparation 2).

## Resource availability

### Lead contact

Further information and requests for resources and reagents should be directed to and will be fulfilled by the lead contact, Maria-Elena Torres-Padilla (torres-padilla@helmholtz-muenchen.de).

### Technical contacts

Technical questions on executing this protocol should be directed to and will be answered by the technical contacts, Diego Rodriguez-Terrones (diego.terrones@imba.oeaw.ac.at) and Marlies E. Oomen (marlies.oomen@helmholtz-munich.de).

### Materials availability

No new materials were generated for this study.

### Data and code availability

Code can be found on GitHub (https://github.com/meoomen/Smartseq5). Data used in this study were originally generated for Oomen, Rodriguez-Terrones et al.^1^ and can be accessed through the GEO project series number GSE225056.

## Acknowledgments

Work in the M.E.T.-P. laboratory is funded by the 10.13039/501100009318Helmholtz Association, the 10.13039/501100001659German Research Foundation (10.13039/501100001659DFG) Project-ID 213249687 (SFB 1064), and the NIH 4DN program (grant no. 5U01DK127391-03). M.E.O. was a recipient of an 10.13039/100004410EMBO long-term fellowship (ALTF 91-2021).

## Author contributions

D.R.-T. performed experiments and M.E.O. performed most of the computational work. D.R.-T., M.E.O., and M.E.T.-P. contributed to the experimental design. The manuscript was prepared, proofread, and approved by all authors.

## Declaration of interests

M.E.T.-P. is a member of the ethics advisory panel of MERCK.

## Supplemental information

Table S1. Table containing custom oligo sequences suggested for use in the Smart-seq+5' protocol

## References

[1] Oomen M.E., Rodriguez-Terrones D., Kurome M., Zakhartchenko V., Mottes L., Simmet K., Noll C., Nakatani T., Mourra-Diaz C.M., Aksoy I. (2025). An atlas of transcription initiation reveals regulatory principles of gene and transposable element expression in early mammalian development. Cell.

[2] Lanciano S., Cristofari G. (2020). Measuring and interpreting transposable element expression. Nat. Rev. Genet..

[3] Takahashi H., Lassmann T., Murata M., Carninci P. (2012). 5′ end–centered expression profiling using cap-analysis gene expression and next-generation sequencing. Nat. Protoc..

[4] Shiraki T., Kondo S., Katayama S., Waki K., Kasukawa T., Kawaji H., Kodzius R., Watahiki A., Nakamura M., Arakawa T. (2003). Cap analysis gene expression for high-throughput analysis of transcriptional starting point and identification of promoter usage. Proc. Natl. Acad. Sci. USA.

[5] Carbonell-Sala S., Perteghella T., Lagarde J., Nishiyori H., Palumbo E., Arnan C., Takahashi H., Carninci P., Uszczynska-Ratajczak B., Guigó R. (2024). CapTrap-seq: a platform-agnostic and quantitative approach for high-fidelity full-length RNA sequencing. Nat. Commun..

[6] Plessy C., Bertin N., Takahashi H., Simone R., Salimullah M., Lassmann T., Vitezic M., Severin J., Olivarius S., Lazarevic D. (2010). Linking promoters to functional transcripts in small samples with nanoCAGE and CAGEscan. Nat. Methods.

[7] Islam S., Kjällquist U., Moliner A., Zajac P., Fan J.B., Lönnerberg P., Linnarsson S. (2011). Characterization of the single-cell transcriptional landscape by highly multiplex RNA-seq. Genome Res..

[8] Islam S., Kjällquist U., Moliner A., Zajac P., Fan J.B., Lönnerberg P., Linnarsson S. (2012). Highly multiplexed and strand-specific single-cell RNA 5′ end sequencing. Nat. Protoc..

[9] Kouno T., Moody J., Kwon A.T.J., Shibayama Y., Kato S., Huang Y., Böttcher M., Motakis E., Mendez M., Severin J. (2019). C1 CAGE detects transcription start sites and enhancer activity at single-cell resolution. Nat. Commun..

[10] Rodriguez-Terrones D. (2019).

[11] Islam S., Zeisel A., Joost S., La Manno G., Zajac P., Kasper M., Lönnerberg P., Linnarsson S. (2013). Quantitative single-cell RNA-seq with unique molecular identifiers. Nat. Methods.

[12] Soumillon M., Cacchiarelli D., Semrau S., van Oudenaarden A., Mikkelsen T.S. (2014). Characterization of directed differentiation by high-throughput single-cell RNA-Seq. bioRxiv.

[13] Bagnoli J.W., Ziegenhain C., Janjic A., Wange L.E., Vieth B., Parekh S., Geuder J., Hellmann I., Enard W. (2018). Sensitive and powerful single-cell RNA sequencing using mcSCRB-seq. Nat. Commun..

[14] Hashimshony T., Wagner F., Sher N., Yanai I. (2012). CEL-Seq: Single-Cell RNA-Seq by Multiplexed Linear Amplification. Cell Rep..

[15] Hashimshony T., Senderovich N., Avital G., Klochendler A., de Leeuw Y., Anavy L., Gennert D., Li S., Livak K.J., Rozenblatt-Rosen O. (2016). CEL-Seq2: Sensitive highly-multiplexed single-cell RNA-Seq. Genome Biol..

[16] Macosko E.Z., Basu A., Satija R., Nemesh J., Shekhar K., Goldman M., Tirosh I., Bialas A.R., Kamitaki N., Martersteck E.M. (2015). Highly parallel genome-wide expression profiling of individual cells using nanoliter droplets. Cell.

[17] Hagemann-Jensen M., Ziegenhain C., Chen P., Ramsköld D., Hendriks G.J., Larsson A.J.M., Faridani O.R., Sandberg R. (2020). Single-cell RNA counting at allele and isoform resolution using Smart-seq3. Nat. Biotechnol..

[18] Hagemann-Jensen M., Ziegenhain C., Sandberg R. (2022). Scalable single-cell RNA sequencing from full transcripts with Smart-seq3xpress. Nat. Biotechnol..

[19] Hahaut V., Pavlinic D., Carbone W., Schuierer S., Balmer P., Quinodoz M., Renner M., Roma G., Cowan C.S., Picelli S. (2022). Fast and highly sensitive full-length single-cell RNA sequencing using FLASH-seq. Nat. Biotechnol..

[20] Hermant C., Mourra-Díaz C.M., Oomen M.E., Altamirano-Pacheco L., Pal M., Nakatani T., Torres-Padilla M.-E. (2025). The transcription factor SRF regulates MERVL retrotransposons and gene expression during zygotic genome activation. Genes Dev..

[21] Olszańska B., Borgul A. (1993). Maternal RNA content in oocytes of several mammalian and avian species. J. Exp. Zool..

[22] Olds P.J., Stern S., Biggers J.D. (1973). Chemical estimates of the RNA and DNA contents of the early mouse embryo. J. Exp. Zool..

[23] Bachvarova R., De Leon V. (1980). Polyadenylated RNA of mouse ova and loss of maternal RNA in early development. Dev. Biol..

[24] Liao Y., Smyth G.K., Shi W. (2014). featureCounts: an efficient general purpose program for assigning sequence reads to genomic features. Bioinformatics.

[25] Ramírez F., Ryan D.P., Grüning B., Bhardwaj V., Kilpert F., Richter A.S., Heyne S., Dündar F., Manke T. (2016). deepTools2: a next generation web server for deep-sequencing data analysis. Nucleic Acids Res..

[26] Jin Y., Tam O.H., Paniagua E., Hammell M. (2015). TEtranscripts: a package for including transposable elements in differential expression analysis of RNA-seq datasets. Bioinformatics.

